# Oxidative Stress and Epigenetic Regulation in Ageing and Age-Related Diseases

**DOI:** 10.3390/ijms140917643

**Published:** 2013-08-28

**Authors:** Chiara Cencioni, Francesco Spallotta, Fabio Martelli, Sergio Valente, Antonello Mai, Andreas M. Zeiher, Carlo Gaetano

**Affiliations:** 1 Division of Cardiovascular Epigenetics, Department of Cardiology, Goethe University, Frankfurt am Main 60596, Germany; E-Mails: pitini@hotmail.it (C.C.); fspallotta@gmail.com (F.S.); 2 Molecular Cardiology Laboratory, IRCCS-Policlinico San Donato, San Donato Milanese, Milan 20097, Italy; E-Mail: fabio.martelli@grupposandonato.it; 3 Pasteur Institute-Cenci-Bolognetti Foundation, Department of Drug Chemistry and Technologies, Sapienza University of Rome, Rome 00185, Italy; E-Mails: sergio.valente@uniroma1.it (S.V.); antonello.mai@uniroma1.it (A.M.); 4 Internal Medicine Clinic III, Department of Cardiology, Goethe University, Frankfurt am Main 60596, Germany; E-Mail: zeiher@em.uni-frankfurt.de

**Keywords:** epigenetics, ageing, oxidative stress, cardiovascular, endothelial, cardiac

## Abstract

Recent statistics indicate that the human population is ageing rapidly. Healthy, but also diseased, elderly people are increasing. This trend is particularly evident in Western countries, where healthier living conditions and better cures are available. To understand the process leading to age-associated alterations is, therefore, of the highest relevance for the development of new treatments for age-associated diseases, such as cancer, diabetes, Alzheimer and cardiovascular accidents. Mechanistically, it is well accepted that the accumulation of intracellular damage determined by reactive oxygen species (ROS) might orchestrate the progressive loss of control over biological homeostasis and the functional impairment typical of aged tissues. Here, we review how epigenetics takes part in the control of stress stimuli and the mechanisms of ageing physiology and physiopathology. Alteration of epigenetic enzyme activity, histone modifications and DNA-methylation is, in fact, typically associated with the ageing process. Specifically, ageing presents peculiar epigenetic markers that, taken altogether, form the still ill-defined “ageing epigenome”. The comprehension of mechanisms and pathways leading to epigenetic modifications associated with ageing may help the development of anti-ageing therapies.

## 1. Introduction

**Figure 1:**
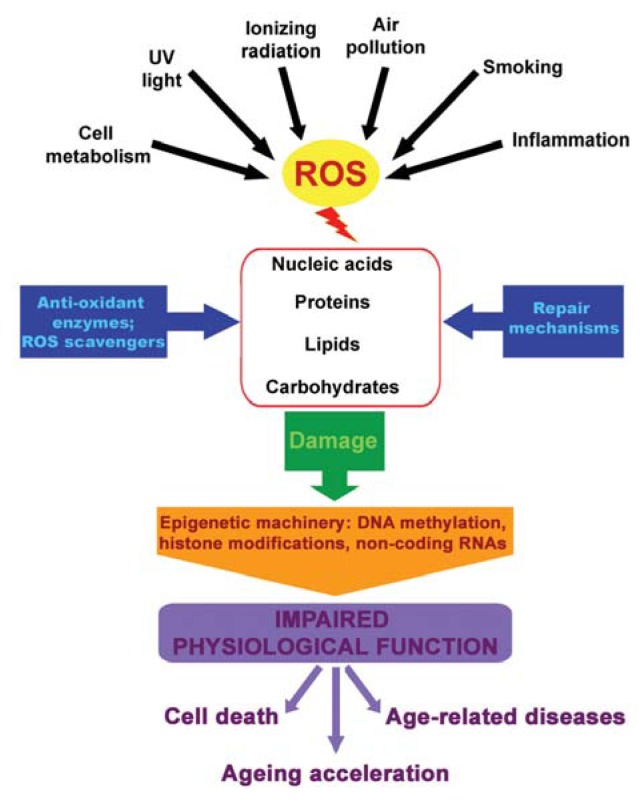
Oxidative stress, epigenetics and ageing. ROS, reactive oxygen species.

Ageing is a multidimensional irreversible accumulation of physical, environmental and social changes. Nowadays, ageing biology and pathobiology are emerging as one of the most compelling areas of biomedical research, owing to current demographic trends and associated healthcare costs in Western societies [1,2]. Both the exponential growth of the literature on ageing during the last few years and the new “-omic” technology development for the study of lifespan revealed a great deal of interest in ageing and ageing-associated diseases among a large number of academic scientists and industrial entities [1]. From the onset of reproductive maturity, throughout the organism’s life, the efficiency of various physiological processes progressively declines [3,4]. The gradual loss of homeostatic mechanisms associated with ageing is hypothetically due to an accumulation of molecular oxidative damage [5–7]. Indeed, the “Free Radical Theory of Ageing” is based on oxygen toxicity [5,8]. Molecular oxygen is a bi-radical able to generate partially reduced molecules and, then, reactive oxygen species (ROS). ROS can be detoxified within the cell by several kinds of antioxidants, based on both enzymatic and non-enzymatic mechanisms [5,8–10]. Examples of intracellular antioxidant enzymes are: superoxide dismutase (SOD), catalase, glutathione peroxidase, peroxiredoxin and sulfiredoxin [5,8,10,11], whereas examples of low molecular weight antioxidants are: glutathione, vitamin C, vitamin A and vitamin E [5,8,10,11]. When all these endogenous antioxidants are insufficient, ROS increase, altering the cell normal redox state and, thus, provoking oxidative stress. High ROS levels cause toxic effects in the cell, because they are potentially detrimental for biological macromolecules, such as lipids, nucleic acids and proteins (Figure 1) [5,9]. In mammalian cells, ROS are mainly produced during physiological processes, such as cellular respiration, the activation of the arachidonic acid cascade and by several enzymes, including, for example, cytochrome p450, Nicotinamide Adenine Dinucleotide (NADH)/Nicotinamide Adenine Dinucleotide Phosphate (NADPH) oxidase and nitric oxide synthase [5,9].

As a consequence of ROS accumulation, oxidative stress, the imbalance of the normal redox state, increases exponentially with age, paralleled by a remarkable decline of the cell repair machinery [10] (Figure 1). Oxidative stress contributes to the pathogenesis of several cardiovascular, pulmonary and neuronal disorders common among elderly people, such as myocardial infarction, diabetes, atherosclerosis, chronic obstructive pulmonary disease (COPD) or Alzheimer’s disease [11–13].

Very recent studies indicated the multifactorial etiology of ageing-associated diseases as related to both genetic and epigenetic changes in the genome [14]. Although at the very beginning, scientists focused primarily on the genetic component of ageing, we would like to stress here that epigenetic mechanisms involved during ageing may play important physiopathological roles above all in the presence of oxidative stress (Figure 1).

## 2. The Epigenetic Machinery

Epigenetics studies the somatically-acquired and, in some cases, trans-generationally inherited modifications of chromatin able to alter gene expression without changing the DNA blue-print [15,16]. Epigenetic mechanisms may act both qualitatively, to induce flexible, short-term gene silencing (histone tail modifications), and quantitatively, to provoke more stable, long-term gene expression (DNA methylation) [15]. The so-called epigenome control, in fact, relies on a large number of histone-modifying complexes, DNA methylation enzymes and non-coding RNAs, which, to a different extent, regulate chromatin structure [17].

Histones can be modified at many sites where the principal covalent modifications are: acetylation, phosphorylation, methylation, isomerization, ubiquitination and sumoylation (see, for review, [17]). Modified histone residues constitute the docking site for distinct chromatin-binding proteins, which direct the dynamic transition between transcriptionally active (euchromatin) and transcriptionally silent (heterochromatin) chromatin as a consequence of the covalent modification to which they are bound. The reversible nature of histone modifications accounts for the presence of chromatin remodeling enzymes with opposite functions, which allow chromatin to have a dynamic structure: for example, the histone acetyltransferases (HATs) and their counterpart, the histone deacetylases (HDACs), or the histone methyltransferases (HMTases), and their opposite, the histone demethylases [17].

DNA methylation is a very important epigenetic modification of cytosine residues in the primary DNA sequence. It is used by the cell as an epigenetic signal to lock genes in the so-called “off” position. Methylation plays an important role during numerous processes, including embryonic development, genomic imprinting, X-chromosome inactivation and the preservation of chromosome stability [18,19]. Specifically, during DNA methylation, a methyl group is added to the carbon-5 position of the cytosine pyrimidine ring by DNA methyltransferases to form 5-methylcytosine (5-MeC) [18,19]. DNA methylation occurring at promoter regions typically represses gene transcription by maintaining chromatin in a closed state [18,19]. This is achieved by recruiting methyl-CpG-binding-domain protein complexes that also contain HDACs. These complexes remove acetyl groups from the histone’s *N*-terminal ends and keep the chromatin in a closed configuration inaccessible to transcription factors and co-activators [18,20]. In contrast, the absence of 5-MeC at un-methylated promoters permits acetylation of histones (via HATs), which, in turn, allows a number of transcription activator complexes [20] to directly access chromatin and to promote transcription of a specific genomic region.

Non-coding RNAs, such as microRNAs, small interfering RNAs and long-non-coding RNAs, represent an additional layer of epigenetic control of gene expression [21]. They play a pivotal role in the regulation of gene transcription, through the recruitment of chromatin modification complexes, including the polycomb group complex [21].

## 3. Epigenetic Traits of Ageing

The entire epigenetic machinery, hitting specific targets and markers, might orchestrate cellular and organismal homeostasis. Alteration of epigenetic mechanisms may lead to accumulation of functional errors and to ageing-associated diseases, such as cancer. Indeed, aged organisms present a peculiarly modified epigenome (Table 1).

### 3.1. Chromatin Alterations

A large body of literature shows that the global hypomethylation occurring in an aged genome is often associated with a decrease in the activity of DNA methylation enzymes [22] with some peak of hyper methylation in specific gene loci, such as *c-fos* [23], *IGF-II* [24] and *p16ink4a* [25]. Furthermore, ageing is characterized by specific histone modifications (Table 1). Histone acetylation on lysine 16 of histone H4 (H4K16) increases gradually, due to a reduction of sirtuin 1 (SIRT1) deacetylase protein level [26–28]. The histone methylation pattern is also sensitive to age: methylation of histones H3 and H4 changes, and depending on residues, it may decrease or increase [29]. The most relevant modified residues affected by the ageing-dependent decrease of the methylation state are: the tri-methylated lysine 36 of histone H3 (H3K36me3), the tri-methylated lysine 9 of histone H3 (H3K9me3) and the mono-methylated lysine 20 of histone H4 (H4K20me) [30]. Among residues affected by an increase of methylation, there are: the tri-methylated lysine 27 of histone H3 (H3K27me3) [30], the mono-/di-methylated lysine 79 of histone H3 (H3K79me/me2) [30] and the tri-methylated lysine 20 of the histone H4 (H4K20me3) [31,32]. Histone modification alterations are linked to changes in the expression level of epigenetic enzymes. Specifically, the, *i.e.*, two histone methylation complexes, the polycomb repressive complex member EZH2 (PRC2) and the polycomb repressive complex member Bmi1 (PRC1) [26], decrease with age, whereas the histone demethylase jumonji domain containing 3 (JMJD3) increases (see Table 1) [33,34].

Senescence-associated heterochromatin foci (SAHFs) are one of cellular senescence markers more easily detectable in mice at the chromatin level. SAHFs are DNA domains that may be recognized when densely stained by 4′,6-diamidino-2-phenylindole (DAPI) (Table 1) [35,36]. These peculiar heterochromatin structures increase in stress-induced senescent cells, where the activation of the cell cycle control Rb-p16^ink4a^ pathway contributes to inducing cell growth arrest. In addition, SAHFs associate with regions of transcriptional repression in which H3K9me3 accumulates [35,37]. The presence of SAHFs seem to play a causal role in cellular senescence, because they induce repression of the E2F transcription factor family, fundamental for the progression of the cell cycle, and cause interruption of cell cycle progression [35].

### 3.2. miRNA Role in Ageing

Several miRNA clusters are up- or down-modulated in different tissues during ageing and are able to hit molecular targets that regulate lifespan, such as insulin-like growth factor 1 (IGF1)/insulin [45], forkhead box, sub-group O (FOXO) [46], SIRT1 [47] and cyclin-dependent kinase inhibitor 1A (p21) (Table 1) [48]. Among miRNAs that affect longevity, in *C. elegans*, micro-RNA 71 (miR-71) acts to increase resistance to heat shock and oxidative stress [38]. Alteration in micro-RNA expression may be involved in the age-associated impairment of organ function often seen in elderly people. The vascular impairment observed during ageing, in fact, is often combined with the altered expression of several micro-RNAs, such as miR-29 [39–41], miR-34a [40–42], miR-217 [40,41] and miR-146 [41,49,50]. miR-29 is upregulated by transcriptional and post-transcriptional mechanisms seen in cultured senescent endothelial cells [40,41] and in old mouse aortas, determining the reduction of extracellular matrix deposition and aneurysm formation [39]. miR-34a has been found upregulated both *in vitro* and *in vivo*, associated with the inhibition of cell proliferation, with a subsequent induction of cellular senescence and premature death, both in endothelial progenitor and mature cells [40,41,43]. In our studies about the effect of oxidative stress on human umbilical vein endothelial cells (HUVECs), we found that ROS induce expression of miR-200 family members [44]. Specifically, we observed that the increase in miR-200c expression upon oxidative stress determined the down-modulation of the zinc finger E-box binding homeobox 1 (Zeb1) transcription factor paralleled by apoptosis and senescence [44].

Cardiac ageing is characterized by cardiomyocyte cell death, hypertrophy and fibrosis, which is also regulated by miRNA alteration. Recently, Boon and coworkers demonstrated the contributive role of miR-34a in the age-dependent decline of cardiac function [42]. Specifically, they found that miR-34a is upregulated in the heart during ageing, determining, *via* repression of its target, PNUTS, telomere erosion, DNA damage and cardiomyocytes apoptosis [42]. The authors further demonstrated that the miR-34a-PNUTS axis rules ischemia reperfusion injury after acute myocardial infarction, a phenomenon strictly associated with oxidative stress damage [42].

## 4. ROS, Epigenetics and Diseases

Cardiovascular diseases are by far the leading cause of morbidity and mortality in industrialized nations [51]. Due to remarkable progress in prevention and acute cardiac patient care, cardiovascular diseases nowadays manifest significantly later in life [51]. Therefore, the incidence of coronary artery disease, myocardial infarction and heart failure, often strictly interconnected, increases almost exponentially with age [51]. Ageing affects cardiovascular tissues, introducing typical markers: aged hearts show hypertrophy and fibrosis, whereas the aged vasculature is affected by arterial thickening and increased stiffness [52]. In this light, the health of cardiac and arterial systems is not mutually exclusive, as each system greatly affects the other [52]. For instance, an increase in arterial stiffness leads to compensatory mechanisms by the myocardium, which includes left ventricular hypertrophy and fibroblast proliferation [53]. Therefore, physiological modifications may determine age-related physiopathological changes, such as vascular dysfunction or insufficient vascular growth and remodeling (hypertension). Heart fibrosis and hypertrophy induce slow propagation of electric impulse throughout the heart, modifying heart rate and the electrical impulse conduction, which increases the incidence of arrhythmias [54]. At the molecular level, ageing is associated with changes in the activity of a series of enzymes necessary for cardiovascular homeostasis. For example, aged endothelial cells exhibit a decrease in endothelial nitric oxide synthase (eNOS) activity and nitric oxide (NO) production [53]. NO is a gaseous molecule able to regulate vasodilatation, shear stress and vascular tone and to prevent thrombotic events and vascular inflammation [55].

The production of ROS increases during ageing and determines oxidative stress, which might be responsible for the SIRT1, a class III histone deacetylases, decreased activity and protein levels [56]. SIRT1 antagonization is involved in senescence of mouse fibroblasts, human cancer cells and endothelial cells [57]. Specifically, Ota and co-workers [57] found that SIRT1 chemical inhibition by sirtinol, or genetically by siRNA gene knockdown, induces a senescence-like phenotype in HUVECs. Specifically, SIRT1 inhibition determines an increase of p53 acetylation with a consequent growth arrest of endothelial cells. On the other hand, SIRT1 overexpression in HUVECs prevented premature senescence in the presence of high levels of hydrogen peroxide (H_2_O_2_). Therefore, SIRT1 results play a pivotal role in the modulation of stress stimuli, at least, in part, via p53 deacetylation [58].

**Figure 2:**
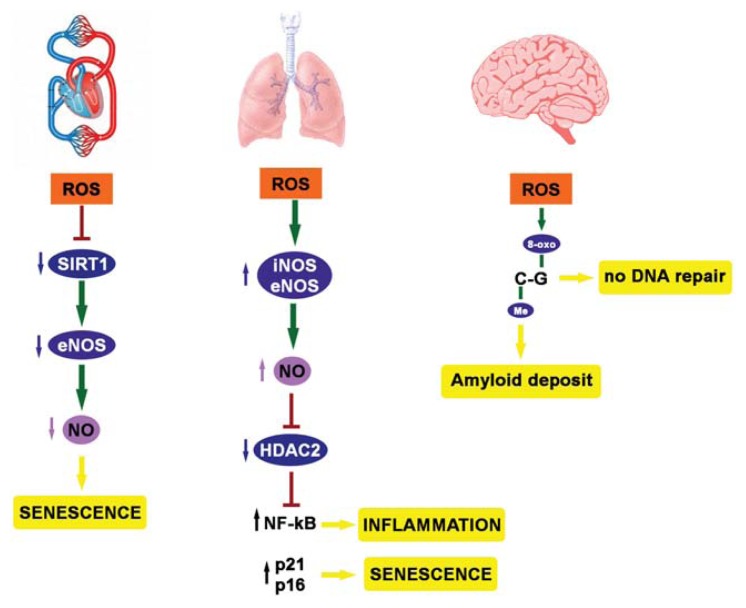
Oxidative stress, epigenetics and diseases. iNOS, inducible nitric oxide synthase; eNOS, endothelial nitric oxide synthase.

Endothelial cell senescence is associated with endothelial dysfunction and vulnerability to atherosclerotic lesions. As mentioned above, NO is fundamental for endothelial function. In line with this observation, Ota *et al.* [59] demonstrated that treatment with cilostazol, a phosphodiesterase 3 (PDE3) inhibitor, induced NO production, thanks to an increased level of cyclic adenosine monophosphate (cAMP) and a consequent eNOS phosphorylation by cAMP/cAMP dependent protein kinase (PKA) and phosphatidylinositol-4,5-bisphosphate 3-kinase (PI3K)/protein kinase B (Akt) signaling pathways. The increase in NO levels may, in turn, enhance SIRT1 activity, which, once more, may delay endothelial senescence [59]. Summarizing, during ageing, oxidative stress accumulates, paralleled with a decrease in NO production, which might be responsible for SIRT1 inactivation. This negative loop facilitates the senescence-like phenotype of endothelial cells (Figure 2). Indeed, it has been recently demonstrated that statins, which induce eNOS activity via SIRT1 upregulation, may inhibit oxidative-dependent endothelial senescence [60].

Besides the cardiovascular system, lungs are exposed to either endogenous or exogenous sources of oxidants. The endogenous oxidants predominantly derive from mitochondrial respiration and phagocyte activation, whereas important exogenous determinants of oxidation are air pollutants, noxious gases and, last, but not least, the smoke of cigarettes [61,62]. The accumulation of ROS directly impairs the function of lung cells, determining posttranslational modifications of histones and non-histone proteins, as well as that of chromatin remodeling enzymes [61,62]. The lung disease in which all these mechanisms are the most evident is chronic obstructive pulmonary disease (COPD) characterized by chronic low-grade systemic inflammation and premature ageing, the so-called “inflamm-ageing”, which determines the obstruction of lung airflow and decreases respiratory function [63]. In these patients, inflammation and cellular senescence are exacerbated by tobacco smoke, which accelerates or induces premature lung ageing [63–65]. Indeed, cigarette smoke contains a number of free radicals and chemical compounds, representing the major source of inhaled ROS, able to alter the intracellular balance between acetylation/deacetylation and methylation/demethylation processes, leading to a deregulated expression of proinflammatory genes [64,65]. Specifically, it has been recently found that cigarette smoke post-translationally modifies histone deacetylase 2 (HDAC2), a class I histone deacetylase, causing a significant reduction in its enzymatic activity [64]. Adenuga *et al.* [66] observed a smoke-dependent HDAC2 inactivation by phosphorylation at Ser394, Ser411, Ser422 and Ser424 in macrophages, human bronchial and primary small airway lung epithelial cells and, *in vivo*, in the mouse lung. In this context, it is the caseine protein kinase 2 (CK2) that induces HDAC2 phosphorylation, leading to its inactivation by ubiquitination and degradation via the proteasome pathway. This physiopathological condition is associated with severe unfavorable effects, such as steroid resistance and abnormal inflammation [66]. Besides phosphorylation, HDAC2 can be modified and inactivated by smoke-induced carbonyl stress and NO-dependent *S*-nitrosylation at cysteine [67,68]. Indeed, in the mouse lung, tobacco smoke increases inducible NOS (iNOS) and eNOS expression and function, respectively, with a consequent increment of NO generation [69]. In this regard, we reported that a deregulated NO synthesis in mice expressing a constitutively active form of eNOS leads to *S*-nitrosylation of HDAC2, with a subsequent loss of its deacetylase activity [70]. A decreased HDAC2 activity has been associated with inflammation and senescence in COPD patients via the increase of histones H3 and H4 acetylation, the activation of the transcription factor, *nuclear factor of kappa light polypeptide gene enhancer in B-cells 1* (*NF-κB)*, and the unscheduled transcription of proinflammatory genes [66,71,72]. Moreover, HDAC2 activity normally delays cellular senescence by negatively regulating pro-senescent genes, such as *cyclin-dependent kinase inhibitor 1A* (*p21*) and *cyclin-dependent kinase inhibitor 2A* (*p16*) [64]. Therefore, a significant reduction in HDAC2 function may accelerate cellular senescence and pulmonary emphysema in COPD patients (Figure 2).

Similarly to the cardiovascular and respiratory systems, the nervous system is also vulnerable to oxidative stress. In fact, although brain holds high concentrations of lipids susceptible to peroxidation and uses high amounts of oxygen to produce energy, it has a relatively deficient anti-oxidant system [73]. Indeed, several lines of evidence have recently underlined the role of oxidative stress and the simultaneous downregulation of antioxidant enzymes during progression from healthy ageing to dementia [74]. Alzheimer’s disease, the typical dementia form of aged people, is characterized by progressive loss of memory and cognitive capacities, due to extracellular amyloid deposits, the so-called senile plaques, and to the formation of intraneuronal aggregates of hyper-phosphorylated tau protein, forming the so-called “neurofibrillary tangles” [74]. Oxidative DNA damage is now accepted as one of the earliest observable events in Alzheimer’s pathogenesis. Remarkably, it can be detected in brains and in peripheral tissues of patients either affected by mild cognitive impairment or at their late stages of Alzheimer’s disease [74]. The most frequent oxidative DNA lesion is the oxidation of guanine to 8-oxo-7,8-dihydro-2′-deoxyguanosine (8-oxo-G), which alters transcription factors binding to DNA as a consequence of a deranged epigenetic signaling [75–77]. Furthermore, astrocytes belonging to the hippocampus and cerebral cortex of Alzheimer’s disease patients often present histone H2A member X phosphorylation, a hallmark of DNA double-strand breaks, which allows formation of intranuclear y-H2AX foci [78]. Of interest, a Cytosine nucleotide next to a Guanine nucleotide arrayed in a linear sequence forms the so-called “CpG island” in specific DNA regions, often intergenic and associated with gene expression control. In this context the presence of oxidized guanosine to form 8-oxo-G, which is one of the most common oxidative DNA damage biomarkers, is often associated with cytosine methylation, leading to the formation of methylated and oxidized CG stretches. These regions might represent sites of interplay between epigenetic and oxidative stress signals potentially relevant in Alzheimer’s disease physiopathology, as proposed by Zawia and coworkers [79]. These authors found that external stimuli during rat brain development might reduce DNA-methyltransferase activity, leading to hypomethylation in the regulatory regions of genes associated with Alzheimer’s disease, such as β-amyloid-precursor-proteins and secretases [79]. In this light, early life exposure to specific stimuli, such as xenobiotic metals, gives an impulse to Alzheimer’s disease, inducing a progressive accumulation of β-amyloid-precursor-proteins and β-amyloids [79]. Coincidently to the formation of these deposits, an increase of cerebral 8-oxo-G levels has been observed [79]. In this way, the epigenetic imprinting can influence the expression of Alzheimer’s disease-related genes, promoting DNA damage and pathogenesis progression. Remarkably, it has been observed that 8-oxo-G cannot be repaired when it is preceded by a methylcytosine [79]. Thus, in the presence of cytosines methylated early in life and belonging to CpG islands, the correction of adjacent guanines in the case of an oxidation event occurring late in life will be prevented, leading to accumulation of oxidative DNA damage in ageing brains (Figure 2) [79]. In conclusion, the authors established that methylation imprinting hits both gene expression and susceptibility to oxidative DNA damage in the late stages of Alzheimer’s disease. Hence, the epigenetic machinery may represent an oxidative stress sensor that orchestrates the progressive homeostasis impairment typical of ageing, thus shaping the cellular senescence often observed during cardiovascular, respiratory and nervous system degeneration.

## 5. Youth Fountain: Struggle with ROS

Lifespan is often correlated to metabolic rate. Albeit that several exceptions exist, it is often observed that the faster the metabolism, the higher the ROS production and, thus, the shorter the lifespan. For this reason, the controlled reduction of oxidative stress may represent a way to slow the progressive homeostasis impairment occurring during ageing. At present, several studies pointed out different methods to increase organism lifespans, including caloric restriction, deletion of p66ShcA and enhancement of SIRT1 activity. All these methods have in common the ability to decrease oxidative stress [10].

Restricted caloric intake significantly modifies the rate of ageing and reduces the age-associated accumulation of oxidized damaged macromolecules [10]. Gene profiles of caloric-restricted aged mice shows low level expression of genes involved in oxidative stress in comparison with aged mice fed ad libitum [6,10]. Thus, caloric restriction prevents several gene expression changes usually occurring in age-related diseases, concurring to prolong animal lifespans by about 20% [10,80].

Sirtuins are the epigenetic beneficial effectors of caloric restriction [81,82]. They belong to a family of seven NAD^+^-dependent class III deacetylases, namely SIRT1-7 [83]. The most characterized component of the family is SIRT1. The aforementioned seems to protect against cardiovascular function impairment common in later ages, cope with stress stimuli and contribute to maintain telomere stability [81,82]. Oxidative stress, in fact, decreases SIRT1 activity to such an extent that some of the negative regulators of oxidative stress, such as p53, forkhead box, sub-group O 3 (FOXO3) and eNOS, become deacetylated and unable to efficiently counteract the progressive homeostasis impairment of endothelial cells [56]. Noteworthy, SIRT1 regulates NO production, contributing indirectly to vascular homeostasis [84]. Specifically, SIRT1 deacetylases eNOS on Lys496 and Lys506 and stimulates its activity [85]. Actually, during ageing, eNOS phosphorylation levels drop, whereas acetylation levels increase. Remarkably, the SIRT1-eNOS coupling not only improves endothelium-dependent vasomotor tone [85,86], but possibly that of a larger number of cell types, as we recently demonstrated in keratinocytes during the skin repair process of mice [87].

Other molecules may have a negative effect on animal lifespan, as in the case of the *p66 Src homology 2 domain-containing* (*p66ShcA*) gene, whose deletion extends life in mice of by least 30% [88]. p66ShcA, together with p46ShcA and p52ShcA, is one of the three isoforms of the mammalian adapter protein, ShcA [89]. All ShcA isoforms contain a common structure, but only p66ShcA presents a unique domain at the *N*-terminus. p52 and p46 are cytoplasmic signal transduction molecules involved in mitogenic signaling from activated tyrosine kinase receptors to Ras, whereas the p66 isoform is devoid of this function and regulates ROS metabolism at the mitochondrial level, promoting oxidative stress in cells and tissues and apoptosis [89]. Epigenetics plays a pivotal role in the regulation of p66ShcA expression [90]. Indeed, p66ShcA expression is partially controlled by different epigenetic modifications of its promoter, which present a high content of CG nucleotides, although not sufficient to qualify as a CpG island-rich region [90]. As previously discussed, DNA methylation of CpG islands is a well-known gene silencing mechanism that confers high stability to chromatin and poor accessibility to transcriptional complexes. p66ShcA promoter analysis in different cell lines showed, in fact, a strong correlation between nucleotides methylation and the expression level of p66ShcA [90]. In cell lines expressing high levels of p66ShcA, bisulfite analysis showed that all the CpG were unmethylated [90]. Conversely, among cell lines not expressing detectable amounts of p66ShcA, the fraction of methylated cytosines ranged between 41% and 100%. In support of DNA methylation as a silencer mechanism for the p66ShcA locus, Ventura and co-workers [90] demonstrated that demethylating treatment of these cell lines induces *de novo* transcription of the *p66ShcA* gene. In addition, further analyses revealed that p66ShcA is transcriptionally repressed by SIRT1, as confirmed by the evidence that p66Shc increases following SIRT1 inhibition. Specifically, SIRT1 directly regulates the p66Shc promoter, decreasing the acetylation of its histone, H3 [91].

Oxidative stress is a determinant of ageing, and p66ShcA knockout results in oxidative stress resistance and low levels of apoptosis. Indeed, murine embryonic fibroblasts derived from p66ShcA knockout (KO) mice are resistant to treatment with oxidant agents and only infrequently respond to oxidative stress stimuli, undergoing apoptosis [88]. On the contrary, murine embryonic fibroblasts overexpressing p66ShcA present an increased level of apoptosis, which correlates with the intracellular production of ROS [88]. In this context, we recently demonstrated that p66ShcA deletion increased both skeletal muscle and endothelial cell resistance to acute ischemia, a tissue injury in which the rapid formation of ROS plays a detrimental role [92]. Intriguingly, we found that p66ShcA not only modulated cell survival, but also differentiation of skeletal muscle progenitors and skeletal muscle regeneration after hind limb ischemia [93]. Moreover, as concerns diabetic injury, in which oxidative stress plays a pivotal role, we reported that the ability of p66ShcA to generate ROS was important for hyperglycemia-sensitivity in bone marrow-derived endothelial progenitor cells and that an active p66ShcA was responsible for the angiogenic impairment induced by diabetes in a mouse model of angiogenesis [94]. In line with this, Chen and coworkers observed the involvement of SIRT1 in diabetic mice [91]. Specifically, SIRT1 was downregulated in the aorta of diabetic mice, and this, in turn, triggered the activation of p66ShcA, causing hyperglycemia-induced endothelial dysfunction [91].

All this evidence shows that p66ShcA may function as a sensor of intracellular concentration of ROS, regulating apoptosis and lifespan. It is well established now that the absence of p66ShcA confers oxidative stress resistance and increases longevity, although this advantage may be limited to the laboratory environment in which animals are kept [95].

## 6. Epigenetic Drugs in Ageing and Age-Related Diseases

Besides genetic interventions, the promise of “healthy ageing” can be pursued, developing epigenetics drugs able to cope with the “aged epigenome”.

The increase of SIRT1 expression and/or activity has positive effects in type 2 diabetes, cancer, cardiovascular diseases, COPD and Alzheimer’s disease [96]. In this light, sirtuin therapeutic activation, by small molecules, is thought to provide a new approach to treat or prevent age-related diseases.

**Figure 3:**
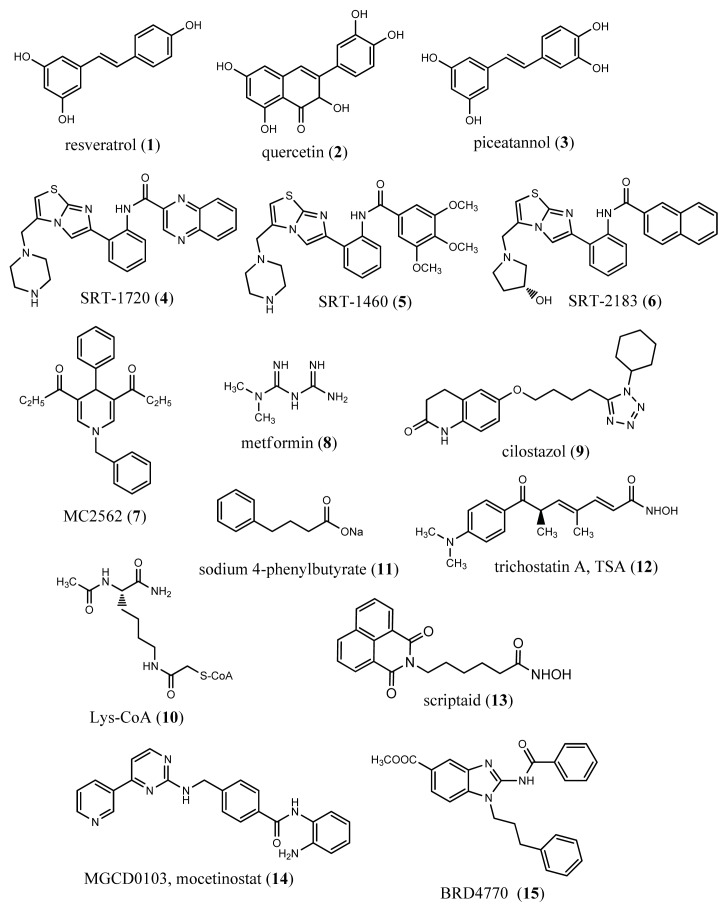
Epigenetic small molecule modulators in ageing and age-related diseases.

Since 2003, resveratrol (see **1** in Figure 3) has been identified as a potent SIRT1 activator that mimics the effect of caloric restriction and regulates longevity in yeast, worms, flies, short-lived fish and mice [96]. In obese rodents, treatment with resveratrol produces a variety of health benefits, including improved metabolic and vascular function, decreased hepatic steatosis, reduced inflammation and improved endurance. Recent clinical studies showed that resveratrol also confers metabolic benefits to humans. In obese humans, one month of resveratrol supplementation, in fact, induced metabolic changes, mimicking the effect of caloric restriction. This beneficial effect has been associated with the positive effect of resveratrol on SIRT1 and the consequent reduction of cellular senescence and inflammation [97]. Resveratrol is currently being evaluated in clinical trials for the treatment of several ageing-related pathologies (see Table 2 for details).

Other epigenetic molecules are now under evaluation for their potentially positive effect in age-associated diseases. Quercetin (**2**), in fact, has been shown to protect against emphysema, a beneficial effect probably due to an increased expression of SIRT1. This observation is in agreement with prior studies about the property of quercetin to activate mammalian SIRT1 or its yeast orthologous Sir2 [98]. Other polyphenolic compounds, including piceatannol (**3**), can also activate SIRT1. Although these compounds have a modest effect on SIRT1, compared to resveratrol, nevertheless, they may have a beneficial application in the treatment of lung inflammation [98].

Several synthetic SIRT1 activators have been recently developed for the treatment of age-associated diseases, including type 2 diabetes [98]. These activators are known as SRT1720 (**4**), SRT1460 (**5**), SRT2183 (**6**), SRT2104 and SRT2379. The most potent among these compounds is SRT1720 (EC_1.5_ = 0.16 μM), which improves glucose homeostasis and insulin sensitivity in animal models of type 2 diabetes [99]. Furthermore, due to SIRT1 activation, it was found that SRT1720 reduced cigarette smoke-induced cellular senescence in the lung. [100]. In addition, SRT1720 improved survival and the health of obese mice [101], suggesting that designing novel molecules that are safe and effective in promoting longevity and preventing multiple age-related diseases in mammals may represent a promising perspective. Of note, some of these compounds are now in phase I/II clinical trials (see Table 2).

In 2009, a new class of 1,4-dihydropyridine derivatives (DHPs) was recognized as a novel SIRT activator (EC_1.5_, SIRT1 = 1 μM), showing a reduction in cellular senescence of primary human mesenchymal stem cells similar to resveratrol. When tested in murine C2C12 myoblast cell line, the most potent compound of this class, diethyl 1-benzyl-1,4-dihydro-4-phenylpyridine-3,5-dicarboxylatenamed (MC2562, **7**), showed a dose-dependent increase in mitochondrial activity with a mechanism involving PGC-1α [102]. *In vitro* and *in vivo* studies revealed that the activation of SIRTs by MC2562 stimulated keratinocyte proliferation via eNOS phosphorylation and NO production, highlighting its effectiveness in accelerating wound repair in a mouse experimental model of skin damage [87].

Metformin (**8**) is a widely used drug for the reduction of hyperglycemia in type 2 diabetes. A recent study demonstrated that the beneficial effect of metformin is associated with the activation and induction of SIRT1 [103]. Further studies revealed that metformin targets AMP-activated protein kinase (AMPK), an upstream kinase important for activating SIRT1. Although the mechanism of metformin action in diabetes, lung inflammation and other ageing-associated diseases remains elusive, clinical trials are ongoing to study the effect of metformin in the treatment of COPD and its complications (Table 2).

Cilostazol (**9**), a selective inhibitor of PDE3, has been reported to protect endothelium, after ischemic damage, through the induction of a significant production of NO [59]. It seems to increase eNOS phosphorylation via a dose-dependent positive effect on SIRT1 expression. The effect of cilostazol on premature senescence is, in fact, abrogated by SIRT1 inhibition [59].

Expression levels of the histone acetyltransferases, p300 and cAMP-responsive element-binding protein-binding protein (CBP), have been reported to decrease with age in mouse models [104]. Remarkably, the genetic or pharmacological inhibition of p300 activity [the latter obtained by using Lys-CoA (**10**), a bi-substrate p300 inhibitor] led to growth inhibition, downregulation of cyclin E and activation of the senescence-associated acidic β-galactosidase in human melanocytes and melanoma cells, whose proliferation often occurs in elderly people [105].

Although HDAC inhibitors (HDACi) are mainly studied for their anti-cancer activity, they also show other biological properties, including anti-inflammatory and neuroprotective ones. In recent years, experimental data emerged on the life-extending potential of synthetic HDACi. A substantial increase in both average and maximum survival without loss of motility, resistance to stress or fertility was observed during feeding *Drosophila melanogaster* with the HDACi, 4-phenylbutyrate (**11**), throughout adulthood [106]. Another study found that also trichostatin A (TSA, **12**), the prototype pan-HDAC inhibitor, significantly extended the lifespan of flies [107]. Further experiments showed that both TSA and phenylbutyrate were to extend *Drosophila* lifespan [108].

*In vivo* studies demonstrated that pan-HDACi can slow or reverse pathological cardiac hypertrophy [109,110]. Treatment with the pan-HDACi TSA, in fact, reduced or prevented the development of cardiac hypertrophy in transgenic mice. TSA treatment was also shown to reverse established cardiac hypertrophy in mice subjected to aortic constriction. Another HDACi, scriptaid (**13**), has been found to be able to blunt cardiac hypertrophy in a pressure-overload mouse model, reducing the size of cardiomyocytes, while improving ventricular performance. In this context, studies performed in genetically engineered mice and isolated cardiomyocytes suggested a role for HDAC2 in heart failure. More definitive answers likely will come from the use of small molecule inhibitors tailored to selected HDAC isoforms. An apicidin derivative (API-D) which is selective predominantly for the class I HDACs, 1, 2 and 3, was shown to effectively suppress cardiac hypertrophy and to improve cardiac performance in the presence of pressure overload [110]. Recently, it has been reported that only the class I HDACi mocetinostat, (MGCD-0103, **14**), and not a class II HDACi, was able to re-express the *dual specificity protein phosphatase 5* (*dusp5*) gene, leading to the inhibition of pro-hypertrophic gene expression. This finding enlightens a potentially novel pathway target of HDACi [111].

A number of histone methyl markers have been reported to be modified with ageing. In general, *in vitro* and *in vivo* studies revealed a global increase in H4K20me3, as well as a decrease of tri-methylated lysine 9 of histone H3 (H3K9me3) and H3K27me3. Interestingly, the Ash-2 complex, which trimethylates H3K4, is a negative regulator of lifespan in *Caenorhabditis elegans* [31–35]. Nevertheless, only one small molecule, the 2-(Benzoylamino)-1-(3-phenylpropyl)-1H-benzimidazole-5-carboxylic acid methyl ester (BRD4770) compound (**15**), has been recently described to inhibit the lysine 9 of histone H3 (H3K9) methyltransferase, G9a, reducing the levels of H3K9me3 and inducing senescence in pancreatic adenocarcinoma PANC-1 cells through activation of ataxia telangiectasia mutated (ATM) kinase [112]. In light of these observations, although the situation is promising, a large amount of work remains to be done in the field of epigenetics to develop effective and enzyme-specific drugs with potential therapeutic application in ageing-associated diseases.

## 7. Concluding Remarks

The accumulation of oxidative stress might orchestrate the progressive homeostasis impairment that leads to the loss of function typical of aged tissues, which often degenerate in severe pathologies, such as coronary artery diseases, Alzheimer’s disease and COPD. Here, we reviewed how epigenetics, using all its “weapons” such as histone-modifying enzymes and DNA-methylation, rules out stress stimuli and identifies part of the mechanisms associated with the physiopathology of ageing-associated diseases. In summary, ageing presents specific epigenetic markers, which, taken altogether, could define the ageing epigenome. These modifications may also be part of a physiopathological processes undergone during the onset of ageing-associated diseases. The next challenge will be the manipulation of this modified epigenome by the use of small molecules: in fact, despite the evidence of a great number of epi-markers, which change during ageing, only a few epi-drugs have been tested in this context, so far. The understanding of epigenetic pathways involved in ageing and ageing-associated diseases cues the development of new therapeutic treatments to contrast relentless tissue impairment, thus promising “healthy ageing”. In this context, we suggest that controlling ROS production may represent the first step towards the achievement of this aim. As described, SIRT1 and p66ShcA, strictly interconnected with each other, might represent two promising targets conferring oxidative stress resistance to target cells and, consequently, delaying organism functional impairment. Although quite a large amount of work is still needed and there is evidence that the above-mentioned targets are not the only ones important in gaining “healthy ageing”, they may represent the beginning of the struggle to control ageing physiology and physiopathology.

## Figures and Tables

**Table 1:** Epigenetic traits of ageing.

Epigenetic ageing marker	Regulation	Reference
Global DNA methylation	Decreased	[22]
DNA methylase activity	Decreased	[22]
PRC1, PRC2	Decreased	[26]
SIRT1	Decreased	[22,26,27]
H3K36me3, H3K9me3, H4K20me	Decreased	[30]
miR-71	Decreased	[38]
*c-fos*, *IGF-II*, *p16Ink4a* methylation	Increased	[23–25]
H4K16ac	Increased	[22,26,27]
JMJD3	Increased	[33,34]
H3K27me3, H3K79me/me2	Increased	[30]
H4K20me3	Increased	[31,32]
SAHFs	Increased	[35–37]
mir-29	Increased	[39–41]
mir-34a	Increased	[40–43]
mir-200 family	Increased	[44]

Notes: PRC1, polycomb-group repressive complex 1; PRC2, polycomb-group repressive complex 2; SIRT1, sirtuin 1; H3K36me3, tri-methylated lysine 36 of histone H3; H3K9me3, tri-methylated lysine 9 of histone H3; H4K20me, mono-methylated lysine 20 of histone H4; miR-71, micro-RNA 71; *c-fos*, FBJ murine osteosarcoma viral oncogene homolog; *IGF-II*, insulin-like growth factor II; *p16Ink4a*, cyclin-dependent kinase inhibitor 2A; H4K16ac, acetylated lysine 16 histone H4; JMJD3, histone demethylase jumonji domain containing 3; H3K27me3, tri-methylated lysine 27 of histone H3; H3K79me/me2, mono-/di-methylated lysine 79 of histone H3; H4K20me3, tri-methylated lysine 20 of the histone H4; SAHFs, senescence-associated heterochromatin foci; miR-29, micro-RNA 29; miR34a, micro-RNA 34a; miR-200, micro-RNA 200 family.

**Table 2:** Epigenetic modulators in clinical trials for age-related diseases.

Drugs	Condition	clinicaltrials.gov Identifier	Phase
Resveratrol	Type 2 diabetes	NCT01677611	I, Completed
Resveratrol	Vascular resistance, aging, hypertension, antioxidants, aerobic capacity	NCT01842399	II
Resveratrol	Healthy	NCT00996229	III
Resveratrol	Alzheimer’s disease	NCT00678431	III, completed
SRT-2104	Type 2 diabetes	NCT00937872, NCT00933062, NCT00933530, NCT01018017	I, II
SRT-2379	Type 2 diabetes	NCT01018628	I
Metformin	COPD	NCT01247870	IV
